# Double-Branch Multi-Scale Contextual Network: A Model for Multi-Scale Street Tree Segmentation in High-Resolution Remote Sensing Images

**DOI:** 10.3390/s24041110

**Published:** 2024-02-08

**Authors:** Hongyang Zhang, Shuo Liu

**Affiliations:** 1 Aerospace Information Research Institute, Chinese Academy of Sciences, Beijing 100094, China; zhanghongyang20@mails.ucas.ac.cn; 2 University of Chinese Academy of Sciences, Beijing 101408, China

**Keywords:** street trees, segmentation, db-msc net, deep learning, neural network, remote sensing images

## Abstract

Street trees are of great importance to urban green spaces. Quick and accurate segmentation of street trees from high-resolution remote sensing images is of great significance in urban green space management. However, traditional segmentation methods can easily miss some targets because of the different sizes of street trees. To solve this problem, we propose the Double-Branch Multi-Scale Contextual Network (DB-MSC Net), which has two branches and a Multi-Scale Contextual (MSC) block in the encoder. The MSC block combines parallel dilated convolutional layers and transformer blocks to enhance the network’s multi-scale feature extraction ability. A channel attention mechanism (CAM) is added to the decoder to assign weights to features from RGB images and the normalized difference vegetation index (NDVI). We proposed a benchmark dataset to test the improvement of our network. Experimental research showed that the DB-MSC Net demonstrated good performance compared with typical methods like Unet, HRnet, SETR and recent methods. The overall accuracy (OA) was improved by at least 0.16% and the mean intersection over union was improved by at least 1.13%. The model’s segmentation accuracy meets the requirements of urban green space management.

## 1. Introduction

Street trees refer to the trees planted on both sides of a road for shade and noise reduction [1]. They are an important part of urban green spaces and urban ecosystems. During the process of street tree management, it is necessary to quickly obtain the information regarding their distribution. There are two traditional ways to obtain this information: one is manual measurement, while the other is using vehicle-mounted LiDAR systems to scan all the street trees along a road [2]. These two methods are inefficient and face difficulty in meeting the requirements of information acquisition speed.

Satellite imaging technology has the advantage of allowing the rapid observation of a large area [3], and satellite remote sensing images are suitable for performing quick segmentation of street trees. However, due to their different species, seasons and growing years [4], street trees show different distribution patterns in satellite images, mainly consisting of concentrated distribution and decentralized distribution [5]. As a result, the sizes of the targets to be segmented are different, so it is necessary to consider both large and small targets in the images used. The methods that researchers have used in their studies in this field are generally based on band calculations, fractional operators, traditional machine learning, etc. [6]. However, these methods cannot demonstrate good performance on both large and small targets. Hong et al. presented two methods of a hierarchical classification technique to distinguish street trees from the neighboring grasslands and roads based on Quickbird images [7]. However, this method easily confused street trees and other trees and missed some small targets, so ensuring its segmentation accuracy proved difficult. Zhao et al. proposed a method for detecting the edges of street trees based on Fourier spectral features [8]. The road center line is pre-extracted using a Gabor filter in frequency domain. Then, the edges of the street trees are extracted according to the buffer of the road center line. The notable problem with this method is that the buffer of the road needs to be set manually. If the buffer is incorrect, the edge of some large targets will be incomplete. Therefore, it is necessary to build a method for the quick segmentation of street trees that considers both large and small targets.

Deep learning has been widely used in many fields, including the segmentation of remote sensing images, because of its excellent performance [9,10]. Since fully convolutional networks (FCNs) first achieved the aim of image segmentation [11], this field has developed rapidly. Many high-performance networks have been proposed, such as Unet [12], the Deeplab series [13], HRnet [14], etc., which have shown good results on different datasets. However, the networks should be improved to consider both small and large targets and adapt to the segmentation of the street trees; their ability to extract multi-scale context features needs to be enhanced. 

In deep neural networks, feature information is extracted by the encoder. The encoder in traditional FCN models mainly extracts the local features of the image [15]. However, due to the limited receptive field of convolutional layers, traditional models are ineffective in extracting larger-scale and global features. Some scholars have proposed the use of pluggable modules in the encoder to improve the feature extraction ability and keep more features from loss [16]. This helps the model retain more features, but it shows little improvement in larger-scale applications. 

The self-attention-mechanism-based Transformer structure has been applied to image segmentation in recent years [17]. The Transformer structure converts images into one-dimensional sequences for input, paying attention to the global features of images at all stages, but not to the local features of images. Therefore, an encoder combined with both an FCN and Transformer can make up for the shortcomings of both and improve the accuracy of street tree segmentation. 

In addition, the brightness of the street trees in the analyzed images differs due to the acquisition time, illumination and other factors, which may reduce the accuracy of multi-scale street tree segmentation. Therefore, it is necessary to introduce other data sources into the segmentation task; some studies have already been carried out in this area [18]. Considering that vegetation index information is easy to obtain from urban green space management, the normalized vegetation index (NDVI) can be input into the network to reduce that impact. The range of the NDVI is [−1, 1], which is different from the RGB bands, so these two parts of information should be input and encoded separately. 

To conquer this problem, in this paper, we proposed a Double-Branch Multi-Scale Contextual Network (DB-MSC Net) for the multi-scale segmentation of street trees from high-resolution remote sensing images. 

The contributions of this paper are as follows:

- The DB-MSC Net is proposed to enhance the ability to segment street trees. The overall accuracy is improved by at least 0.16% and the mIoU is improved by at least 1.13% compared to typical networks.
- We designed a double-branch structure in the network to adapt to the input of both RGB images and the NDVI.
- The MSC block is proposed to improve the ability to extract multi-scale features. It uses a CNN–Transformer hybrid structure to extract both local and global features.

The rest of this paper is organized as follows. Section 2 summarizes the typical segmentation methods based on deep neural networks. In Section 3, the structure of the DB-MSC Net and MSC block is described in detail. Relevant experiments are presented in Section 4. Discussions on our study are outlined in Section 5. Conclusions are provided in Section 6.

## 2. Related Work

In this section, we review several existing, representative methods and improvements for multi-scale segmentation in high-resolution remote sensing images based on deep neural networks. 

There are two main ways to improve multi-scale segmentation ability. One is improving the CNN-based networks, and the other is using Transformer-based networks. 

FCNs were the first networks to realize image segmentation based on deep neural networks. Subsequently, since the precision of multi-scale segmentation is not ideal, researchers began to explore methods of enhancing the ability to extract multi-scale features in neural networks. Chen et al. [19] proposed fully connected CRFs in Deeplab V1 and overcame the localization characteristics of deep neural networks. They proposed Deeplab V2 in 2017 [13], using atrous convolution and a spatial pyramid pooling module to expand the receptive field of the network. In Deeplab V3 and V3+ [20,21], they extended the ASPP module with different dilation rates of atrous convolution, which improved its multi-scale feature extraction ability. In addition, Zhao et al. [22] proposed a pyramid pooling module (PPM), which contains information between different scales and different subregions. Qin et al. [23] proposed an autofocus convolutional layer with parallel convolutional layers possessing different dilation rates and an attention module to learn the weight of different branches, which can adaptively change the size of receptive fields to extract multi-scale features. Gu et al. [24] used two parallel encoders to extract information from different scales and a single decoder to concatenate the information. Tokunaga et al. [25] used three parallel CNNs and weighted concatenation to extract multi-scale information.

The Transformer structure mainly consists of Multi-Head Attention and Feed-Forward Networks [26], showing good performance when extracting global features. Inspired by this advantage, researchers began to improve the Transformer structure for the field of image segmentation. Segmentation Transformer (SETR) [27] first replaced convolutional-layer-based encoders with a pure transformer. The self-attention model is used in global feature learning. Liu et al. [28] proposed the Swin Transformer, which is based on shifted windows to improve computational efficiency and uses hierarchical feature maps to obtain multi-scale feature maps.

In recent years, researchers have conducted many studies on tree extraction using remote sensing images, which can accurately represent the segmentation of street trees. Ye et al. [29] proposed a method of automatically extracting olive crowns, combining RGB images captured by unmanned aerial vehicles (UAVs) and a U^2^-Net neural network, which achieved high accuracy when extracting tree crown numbers in four typical subareas. Zhang et al. [30] proposed a new method for individual tree segmentation and identification based on the improved Mask R-CNN network. Their results showed that this method has more advantages in broadleaf canopy segmentation and number detection. Schürholz et al. [31] applied convolutional neural networks for instance segmentation to accurately delineate individual tree canopies for certain species and identify the area coverage of some mangrove tree species as well as the surrounding land-cover classes. Lv et al. [32] proposed a novel Mask-CSP-attention-coupled network (MCAN) to enhance detail information detection and improve individual tree detection accuracy. The results showed that this network can perform high-precision segmentation in many contexts. Zheng et al. [33] improved the structure of a High-Resolution Network (HR-Net) algorithm to make it more suitable for forest extraction in remote sensing images. However, the networks mentioned above did not focus on the ability to perform multi-scale feature extraction. Liu et al. [34] proposed a Multi-Scale Channel Spatial Attention Network (MSCSA-Net) and improved the overall accuracy of tree segmentation, but this network only used convolutional layers to build the channel and spatial attention module. It did not apply a Transformer structure and its ability to extract global features can be improved. So, it is important to build a CNN–Transformer hybrid network to enhance the ability to conduct global feature extraction. 

In this article, we explore the method of achieving multi-scale street tree segmentation using high-resolution remote sensing images. Our method is a combination of CNN and Transformer which considers both multi-scale local features and global features. The following section provides the architectural details of our proposed model.

## 3. Materials and Methods

### 3.1. Dataset and Data Preprocessing

We proposed a benchmark dataset to test the improvement of our network. The experimental data comprised RGB images and NDVI products from the Beijing-3 International Cooperative (BJ-3N) Satellite. This satellite has an orbital altitude of 620 km and a revisit period of 1 day. The resolution of its panchromatic images is 0.3 m, and the multispectral resolution is 1.2 m. The wavelengths of the spectrum are 450–520 nanometers in blue, 530–590 nanometers in green and 620–690 nanometers in red. 

**Figure 1:**
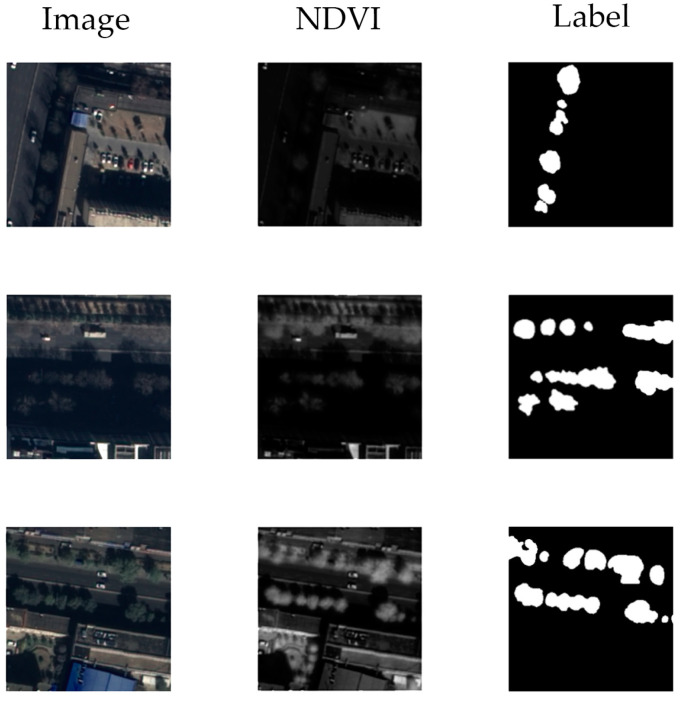
Typical scenarios in the dataset.

Our study used 2 sets of remote sensing products. Each set contained one RGB image without a vegetation index and one NDVI. These two sets of products were both taken over Lhasa, Tibet Autonomous Region, China. One was acquired on 4 November 2021, and the other was acquired on 29 January 2022. We selected 12 street blocks in these two images and marked all the street trees as the label. All the images, NDVI and labels were clipped to fit the size of 256 × 256. We used random shifts, random rotations and horizontal and vertical flips for data augmentation. For RGB images, we used a random brightness jitter of up to ±10%. After that, the dataset was divided into the training set, validation set, and test set in the ratio of 8:1:1. Each set contained pictures with and without street trees. The number of each kind of image in the datasets is shown in Table 1. Figure 1 uses three typical scenarios to show the details of this dataset. The dataset is publicly available on https://pan.baidu.com/s/1xUp1Davs2-58i_oJxGUh7w?pwd=ngg3 (accessed on 25 December 2023). 

### 3.2. The Overall Architecture of DB-MSC Net

**Figure 2:**
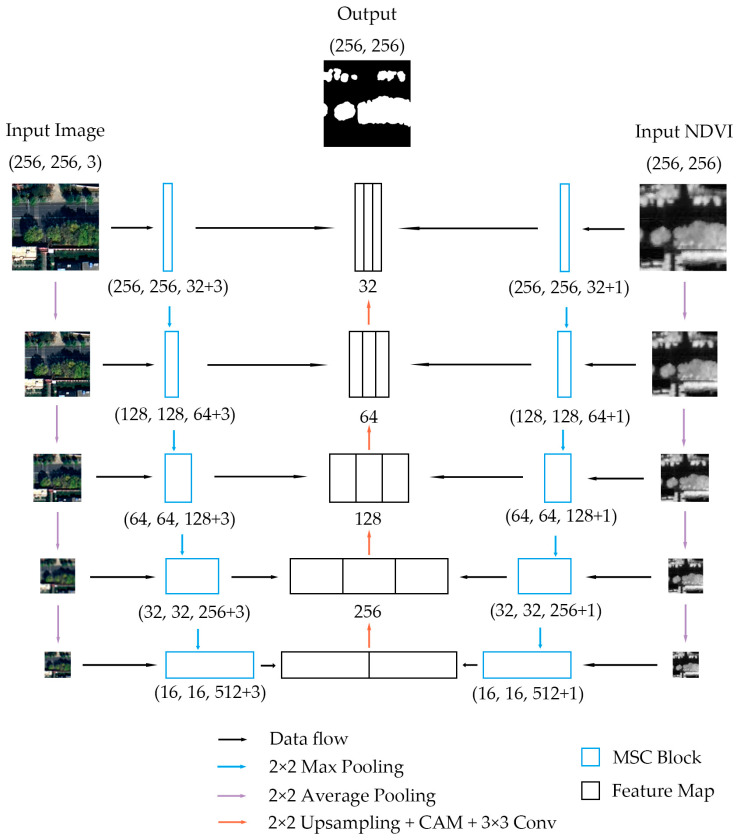
The overall architecture of DB-MSC Net.

The overall architecture of DB-MSC Net is shown in Figure 2. The numbers under each block represent the size or channels of the feature. As shown in the Figure, the DB-MSC Net model has five stages. For the first stage, we designed two input layers to encode image features without vegetation $A_{1}$ and vegetation features $B_{1}$ separately because of the different ranges of RGB images and the NDVI. $A_{1}$ was input into the MSC block at this stage and the output was ${FA}_{1}$. Similarly, the output of $B_{1}$ was ${FB}_{1}$. The process can be expressed by the following formula.

(1) $${FA}_{1}=msc(A_{1},A_{1})$$

(2) $${FB}_{1}=msc(B_{1},B_{1})$$

Starting from the second stage, the input RGB image and NDVI were 2 × 2 average-pooled from the previous stage. The features output from the previous stage, ${FA}_{i-1}$ and ${FB}_{i-1}$, were also 2 × 2 max-pooled and then input into the MSC block together with the image or NDVI. This step is formulated as follows.

(3) $${FA}_{i}=msc\left(Maxpool\left({FA}_{i-1}\right),A_{i}\right)i=2,3,4,5$$

(4) $${FB}_{i}=msc\left(Maxpool\left({FB}_{i-1}\right),B_{i}\right)i=2,3,4,5$$

After encoding, the multi-scale image features ${FA}_{i}$ and vegetation features ${FB}_{i}$ can be extracted. 

In the decoding process, first, ${FA}_{5}$ and ${FB}_{5}$ were concatenated by channel to form the feature $Q_{5}$.

(5) $$Q_{5}=[{FA}_{5},{FB}_{5}]$$

After 2 × 2 bilinear interpolation upsampling, 3 × 3 convolution and ReLU activation function operations for $Q_{5}$, the weight of each channel was calculated and added through the channel attention mechanism (CAM) module to form the feature $P_{4}$. Then, ${FA}_{4}$, $P_{4}$ and ${FB}_{4}$ were concatenated by channel. These operations were then repeated for each stage. This step is formulated as follows.

(6) $$P_{i}=CAM\left(Up\left(Conv(Q_{i+1})\right)\right)i=1,2,3,4$$

(7) $$Q_{i}=\left[{FA}_{i},{P_{i},FB}_{i}\right]i=1,2,3,4$$

The CAM function is defined in Section 3.4. At the end of the model, $Q_{1}$ passes through a 3 × 3 convolutional layer to adjust the number of channels to output the result. 

### 3.3. The Structure of the MSC Block

In DB-MSC Net, we propose a new encoding module, an MSC block, which is important for extracting the multi-scale features of street trees from remote sensing images and vegetation information. This section introduces the detailed structure of this module. 

Taking the MSC block in the middle stage of the network as an example, the input of this module consists of two parts. One is the average pooled RGB image $A_{i}$ or NDVI $B_{i}$, and the other is the max pooled output feature from the MSC block in the previous stage, ${FA}_{i}$ or ${FB}_{i}$. The MSC block extracts the global features from the input image or NDVI and the local feature from the output of the previous MSC block. Finally, the global feature and the local feature are combined to obtain the multi-scale feature as the output of the MSC block. 

The input RGB image or NDVI is first cut into a 16 × 16 size and linear-projected to a 2D matrix with a width of 256. Then, a zero matrix of the same size is initialized and added to that matrix through positional encoding and the matrix x is obtained. x is then mapped to two different subspaces using the learnable matrices $W_{Q}$, $W_{K}$ and $W_{V}$ to obtain the matrices query ($q_{1}$, $q_{2}$), key ($k_{1}$, $k_{2}$) and value ($v_{1}$, $v_{2}$). The formula is as follows.

(8) $$q_{i}=W_{Q}x_{i}\;i=1,2$$

(9) $$k_{i}=W_{K}x_{i}\;i=1,2$$

(10) $$v_{i}=W_{V}x_{i}\;i=1,2$$

Matrix q and the transposition of the matrix k are used for point multiplication. After being multiplied with $d_{q}$, the square root of the dimensions of the matrix k and softmax function, the similarity probability α is obtained. α is multiplied with v to obtain the attention weight matrix y. Finally, after layer normalization, MLP and residual addition, the global feature matrix z is obtained. It is formulated as follows.

(11) $$\alpha_{1,i}=softmax\left(\frac{q_{1}^{T}\cdot k_{i}}{\sqrt{d_{q,k}}}\right)\;i=1,2$$

(12) $$\alpha_{2,i}=softmax\left(\frac{q_{2}^{T}\cdot k_{i}}{\sqrt{d_{q,k}}}\right)\;i=1,2$$

(13) $$y_{1}=\sum_{i=1}^{2}\alpha_{1,i}\cdot v_{i}$$

(14) $$y_{2}=\sum_{i=1}^{2}\alpha_{2,i}\cdot v_{i}$$

The Transformer module is repeated 6 times. After fully extracting global features, the feature is reshaped to the size same as the input image or NDVI and the matrix $F_{0}$ is obtained. 

Matrix ${FA}_{i-1}$ is input to the MSC block after 2 × 2 max pooling. The input matrix first goes through a 5-branch convolution module. The first four convolutional layers have a kernel size of 3 and stride of 1. One convolutional layer is not dilated, and the other 3 convolutional layers have dilation rates of 2, 3 and 5, respectively. The output matrices of each of these four branches, $F_{1}$, $F_{2}$, $F_{3}$ and $F_{4}$, have the same length and width as matrix ${FA}_{i-1}$ and the number of channels is half of ${FA}_{i-1}$. The last convolutional layer has a kernel size of 1 and stride of 1. The output matrix of this layer $F_{5}$ has the same length, same width and twice the channels as ${FA}_{i-1}$. Then, $F_{1}$, $F_{2}$, $F_{3}$ and $F_{4}$ are concatenated by channel and $F_{5}$ is added to obtain local features. Finally, the local and global features are concatenated by channel and the output multi-scale feature ${FA}_{i}$ is obtained. It is formulated as follows.

(15) $${FA}_{i}=\left[\left[F_{1},F_{2},F_{3},F_{4}\right]+F_{5},F_{0}\right]$$

**Figure 3:**
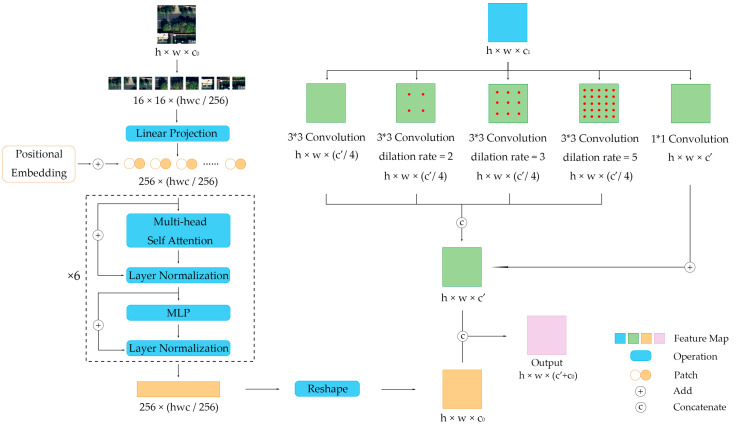
The architecture of the MSC block.

The architecture of MSC block is shown in Figure 3. 

### 3.4. Channel Attention Mechanism (CAM)

**Figure 4:**
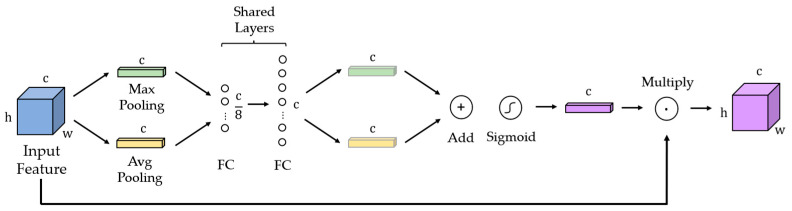
The architecture of the CAM.

The channel attention mechanism (CAM) [35] is used to selectively emphasize informative features while suppressing less-useful features in RGB images and NDVI. Figure 4 shows the architecture of the CAM. 

First, the input feature map is compressed in channel dimensions using max pooling and average pooling to obtain two one-dimensional vectors. Then, the multilayer perceptron (MLP) network is used, and vectors with weight value are generated. The two vectors are added and calculated using the sigmoid function σ. Finally, the vector is multiplied with the input feature and the weighted feature is output, which can be formulated as follows. F is the input feature and $F^{\prime }$ is the output feature.

(16) $$F^{\prime }=CAM\left(F\right)=\sigma (MLP\left(Avgpool\left(F\right)\right)+MLP(Maxpool(F)))$$

### 3.5. Experimental Design and Criteria 

In our study, all experiments were implemented with Tensorflow 2.10.1 on an NVIDIA Geforce RTX 3080 Graphics Processing Unit (GPU) produced by ASUS in Shenzhen, China. CUDA 11.2 toolkit and cuDNN 8.1 were used to speed up the training process. 

The DB-MSC model was compared with FCN-8s [11], Deeplab V3+ [21], PSPNet [22], UNet [12], HRNet [14], SETR [27] and MSCSA-Net [34]. We trained all networks using an Adam optimizer with a beta1 of 0.9, beta2 of 0.999 and adaptive learning rate. We initialized the learning rate at 0.001. The number of overall training iterations was 100 and each GPU batch size is 5. We used the CrossEntropy loss function to calculate the loss of these models. The loss is defined as follows. The experiment was repeated five times and averaged.

(17) $$L=-y_{true}\log\left(y_{pred}\right)-(1-y_{true})log(1-y_{pred})$$

We conducted an ablation study to verify the effects of the improvements on the encoder. Three networks, Double-Branch UNet (DB-UNet), Double-Branch Channel Attention Mechanism UNet (DB-CAM-UNet) and DB-MSC Net, were trained to compare the improvements. The DB-UNet is a double-branch UNet-like network which allows for the separate input of the RGB image and NDVI. The DB-CAM-UNet adds CAM to the decoder based on DB-UNet. 

The training hyperparameters of these three networks were the same as in the contrast experiment. The experiment was repeated five times and averaged.

The results of segmentation can be divided into 4 categories: True Positive (TP) refers to the pixels that are correctly segmented as street trees. False Positive (FP) refers to the pixels that are segmented as street trees but labeled as non-street trees. True Negative (TN) refers to the pixels that are correctly segmented as non-street trees. False Negative (FN) refers to the pixels that are segmented as non-street trees but labeled as street trees. To evaluate our method, four metrics, OA, F1, mIoU and Kappa, were used to evaluate the performance of each segmentation method. OA is the ratio of correctly segmented pixels to the total number of pixels. The F1-score (F1), mIoU and Kappa are defined as follows.

(18) $$OA=\frac{TP+TN}{TP+TN+FP+FN}$$

(19) $$F_{1}=\frac{TP}{2TP+FP+FN}$$

(20) $$mIoU=\frac{TP}{TP+FP+FN}$$

(21) $$p_{0}=TP+TN$$

(22) $$p_{e}=\left(TP+FP\right)\left(TP+FN\right)+\left(FP+TN\right)\left(FN+TN\right)$$

(23) $$Kappa=\frac{p_{0}-p_{e}}{1-p_{e}}$$

## 4. Results

### 4.1. Network Training

**Figure 5:**
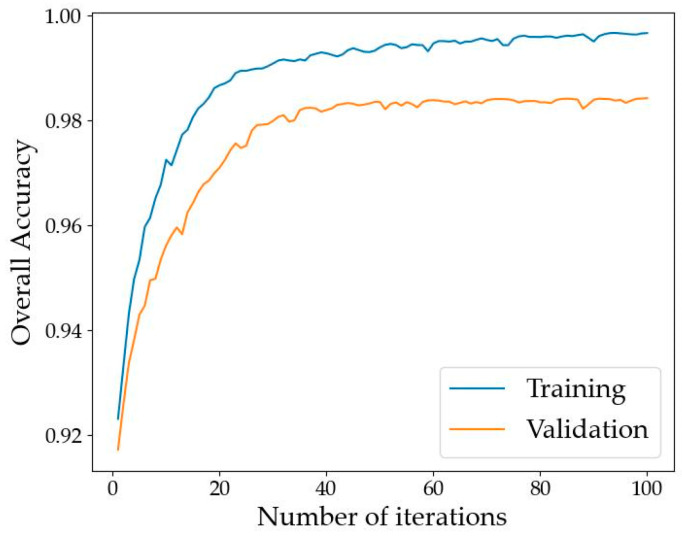
Overall accuracy curves of the DB-MSC Net.

**Figure 6:**
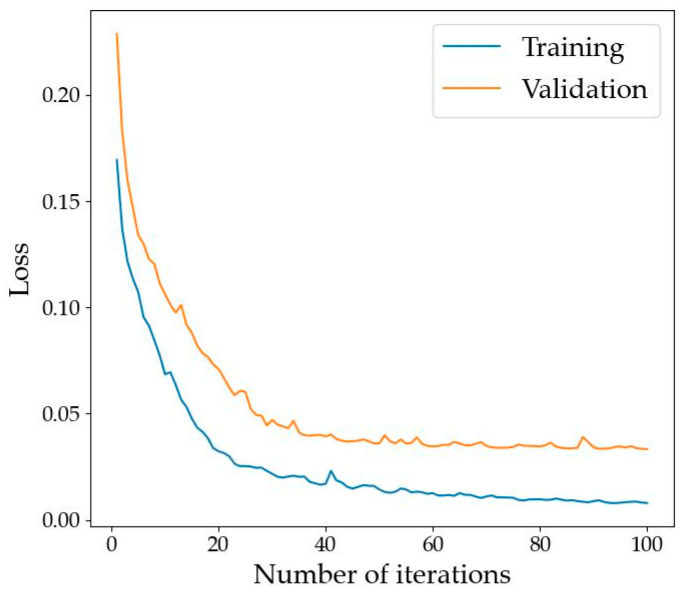
Loss curves of the DB-MSC Net.

The overall accuracy curves of the DB-MSC Net on the training set and verification set are shown in Figure 5. Loss curves of the DB-MSC Net in the training set and verification set are shown in Figure 6. 

Figure 5 and Figure 6 show that the network converged after training on our dataset for 100 epochs.

### 4.2. Street Tree Segmentation

The quantitative results of different networks on our benchmark dataset are shown in Table 2.

The results show that compared to the networks using only convolutional layers like FCN-8s, PSPNet, Deeplab V3+, UNet, HRNet and MSCSA-Net, DB-MSC Net increased the mIoU by at least 1.13%, increased F1 by at least 1.42% and increased Kappa by at least 2.47%. Compared with networks using only Transformer in the encoder, such as SETR, the DB-MSC Net increased the mIoU by 5.11%, increased F1 by 4.57% and increased Kappa by 8.85%.

**Figure 7:**
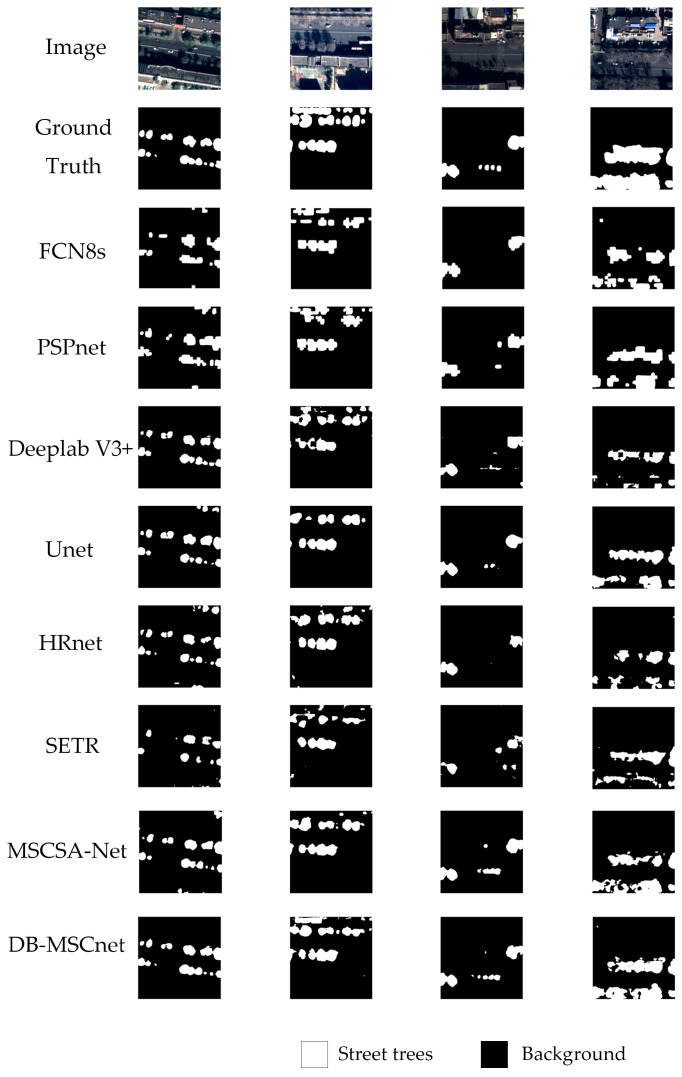
Segmentation results for high-resolution remote sensing images with street trees using different methods.

Four high-resolution remote sensing images with street trees were taken as segmentation examples. Original images, ground truth and segmentation results using FCN-8s, PSPNet, Deeplab V3+, UNet, HRNet, SETR, MSCSA-Net and our DB-MSC Net are shown in Figure 7.

Figure 7 shows that the networks using only convolutional layers under-segmented the large-size street trees. This is more obvious in the last image since the results of these networks are fragmented. The SETR network under-segmented the small-size street trees. In the first three images, it ignored some small street trees. However, the DB-MSC Net was not affected by the different sizes of street trees and showed a good performance. In all these images, the segmentation results of our method were closer to the ground truth. 

### 4.3. Ablation Study

**Figure 8:**
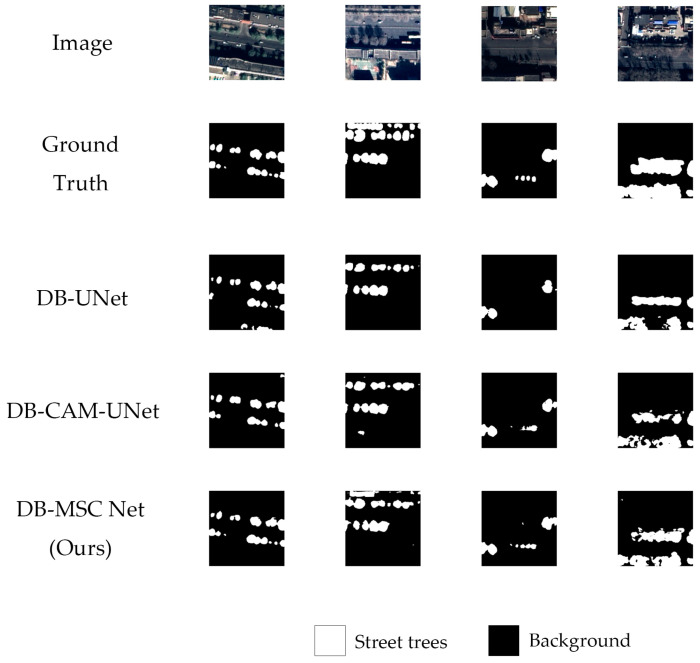
Segmentation results of ablation study.

Table 3 shows the quantitative results of the ablation study, and the segmentation result examples are shown in Figure 8. 

The quantitative results show that compared to the DB-UNet, the DB-CAM-UNet increased the mIoU by 0.19%, increased F1 by 0.92% and increased Kappa by 0.79%. The improvement was not obvious. However, our DB-MSC Net increased the mIoU by 2.33%, increased F1 by 4.53% and increased Kappa by 3.95%, which showed an obvious improvement. 

Figure 8 shows that the DB-UNet under-segmented and over-segmented images. For example, some other trees were incorrectly identified as street trees in the first image. In the other three images, some street trees were not accurately segmented. The DB-CAM-UNet showed some improvements but still under-segmented images. In the second and the fourth image, some large-size street trees were not accurately segmented. By contrast, our DB-MSC Net showed obvious improvements in both large- and small-size street trees. The experiment shows that our improvements to the network were effective. 

## 5. Discussion

There are significant challenges in the segmentation of street trees because of the different sizes of the targets. In this paper, the DB-MSC Net was proposed for the multi-scale segmentation of street trees to improve accuracy. The DB-MSC Net has two branches for the separate input of an RGB image and the NDVI. In the encoding path, MSC blocks were used to replace convolutional blocks. The MSC block combined dilated convolution and Transformer, which enabled the extraction of both local and global features. To further improve the performance of the network, the channel attention model (CAM) was embedded into the decoder. 

Compared to the traditional networks, the overall accuracy of DB-MSC Net showed an improvement of at least 1.13% in mIoU. From the segmentation results, the DB-MSC Net proposed in this paper showed better performance than networks such as FCN-8s, PSPNet, Deeplab V3+, UNet, HRNet, MSCSA-Net and SETR. Street trees of different sizes were accurately segmented. 

The DB-MSC Net accurately segmented multi-scale street trees in high-resolution remote sensing images. However, the method we proposed in this paper did not show a good effect for some street trees in shadow-covered areas. In areas with dense buildings, the segmentation effect was worse due to the shadows of the buildings. This method presents great challenges in segmenting such areas. 

In future work, we will focus on the problem of street tree segmentation in areas covered by the shadows of buildings. We will explore appropriate methods to reduce the effects of shadows.

## 6. Conclusions

This paper focused on the problem of multi-scale segmentation of street trees in high-resolution remote sensing images. We proposed the DB-MSC Net method for performing multi-scale segmentation. The experimental results showed that the proposed method for segmenting street trees in high-resolution remote sensing images had improved accuracy. 

The contrast experiment and ablation study verified the effectiveness of the proposed network by comparing it with two different improved network stages and six typical segmentation models using our BJ-3N satellite image dataset. The results met the accuracy requirements for urban green space management. 

## Figures and Tables

**Table 1:** Number of images in the dataset.

Category	Training Set	Validation Set	Test Set	Total
Contain street trees	7110	885	890	8885
No street trees	890	115	110	1115
Total	8000	1000	1000	10,000

**Table 2:** Performance of different networks.

Model	OA (%)	mIoU (%)	F1 (%)	Kappa (%)
FCN-8s [11]	95.05	63.81	71.13	42.69
PSPNet [22]	95.07	64.62	72.05	44.48
Deeplab V3+ [21]	95.81	69.21	75.98	52.28
UNet [12]	95.98	69.78	76.05	52.43
HRNet [14]	95.44	66.67	73.51	47.50
SETR [27]	94.19	65.84	73.09	46.78
MSCSA-Net [34]	95.87	69.82	76.24	53.16
DB-MSC Net (Ours)	96.14	70.95	77.66	55.63

**Table 3:** Quantitative results of ablation study.

Model	OA (%)	mIoU (%)	F1 (%)	Kappa (%)
DB-UNet	95.65	68.62	73.13	51.68
DB-CAM-UNet	95.87	68.81	74.05	52.47
DB-MSC Net (Ours)	96.14	70.95	77.66	55.63

## Data Availability

Data are contained within the article.
